# Experiences with multiple job holding: a qualitative study among Dutch older workers

**DOI:** 10.1186/s12889-018-5841-7

**Published:** 2018-08-22

**Authors:** S. Bouwhuis, A. De Wind, A. De Kruif, G. A. Geuskens, A. J. Van der Beek, P. M. Bongers, C. R. L. Boot

**Affiliations:** 1 Amsterdam UMC, VU University, Department of Public and Occupational Health, Amsterdam Public Health Research Institute, Van der Boechorststraat 7, 1081 BT Amsterdam, the Netherlands; 2 Netherlands Organisation of Applied Sciences TNO, Schipholweg 77, 2316 ZL Leiden, the Netherlands; 3 Body@Work research center, Van der Boechorststraat 7, 1081 BT Amsterdam, the Netherlands; 4 Department of Health Sciences, Faculty of Science, Amsterdam Public Health Research Institute, Vrije Unviversiteit Amsterdam, De Boelelaan 1085, 1081 HV Amsterdam, The Netherlands

**Keywords:** Multiple job holding, Moonlighting, Dual job holding, Aging employee, Qualitative study, Interviews, Sustainable employability

## Abstract

**Background:**

Multiple job holding (MJH) is a common and growing phenomenon in many countries. Little is known about experiences with MJH among older workers. The objective of the present study is to gain insight in experiences with MJH among Dutch workers aged 45 years and older.

**Methods:**

Multiple job holders were selected from the Study on Transitions in Employment, Ability, and Motivation (STREAM), a Dutch cohort study among persons aged 45 years and older. Purposive sampling was applied to assure heterogeneity regarding gender, educational level, health, financial situation, willingness to continue MJH, and type of MJH (only jobs as employee or also being self-employed). Interviews were conducted until data saturation occurred. Fifteen multiple job holders participated in this study (eight men, seven women). Interviews were digitally recorded, transcribed verbatim and analyzed, along with field notes, using thematic content analysis. The data were openly coded, after which codes were aggregated into themes, which formed a thematic map. In each phase of the analysis at least two researchers were involved to increase reliability.

**Results:**

Experiences with MJH varied from positive to negative. They were influenced by characteristics of individual jobs, e.g. social support at work, as well as characteristics of the combination of jobs, e.g. positive spill-over effects, and conflicts between work schedules. The personal context of multiple job holders, e.g. their age, or reason for MJH, affected how work characteristics influenced experiences. Negative experiences with one job often coincided with negative experience in the other job(s), and problems in the personal context. Some multiple job holders were able to make changes to their situation when desired. For some, this was not possible, which augmented their negative experience.

**Conclusions:**

This study adds to existing knowledge that experiences with MJH are not only influenced by work characteristics but also by the personal context of multiple job holders, and that some workers are able to change their situation when desired, while others are not. Future research should study how different combinations of work and personal characteristics influence sustainable employability of multiple job holders. Policies facilitating life-long learning could increase opportunities to change the MJH situation when desired.

## Background

In many countries, a substantial and growing proportion of the work force holds multiple jobs [1]. Multiple job holding (MJH), also known as dual job holding or moonlighting, is defined as having multiple paid jobs, either as an employee or as being self-employed. It has been associated with higher flexibility of labor markets, higher job mobility and lower job security [2, 3]. Regarding reasons for MJH, previous studies suggest that increasing working hours, and income (security) motivates some workers to have multiple jobs [2, 4, 5]. Other studies suggest that workers have multiple jobs because having heterogeneous jobs can have non-pecuniary benefits [6], such as increased job satisfaction and the acquisition of distinct skills. Previous studies on health consequences of MJH are scarce and have found mixed results. Some studies suggest that multiple job holders have better mental health, especially those who do not have multiple jobs out of financial necessity [7]. On the other hand, multiple job holders sleep less than single job holders and have a higher risk of sustaining injuries [8–10].

More insight into workers’ experiences with MJH may contribute to a better understanding of the relation between MJH, health and sustainable employability more broadly. ‘Experience’ refers firstly to undergoing facts or events, i.e. MJH, and secondly to impressions of and feelings towards such facts or events [11]. Undergoing MJH may be related to health consequences. Previous research on the personal impact of MJH has shown that some multiple job holders experience stress due to conflicting work schedules and long working hours [12]. Long working hours have also been associated with less sleep among multiple job holders [10]. Stress and reduced sleep duration are associated to health problems [13, 14].

Little is known about impressions of MJH, i.e. satisfaction with MJH. However, previous studies have shown that general job satisfaction is associated with health [15]. Satisfaction with MJH may be an important part of general job satisfaction, or it may attenuate or augment the relation between general job satisfaction and health.

Research on experiences with MJH is scarce. To our knowledge, only one Australian qualitative study by Bamberry et al. has examined experiences with MJH [12]. This study found that most workers did not experience MJH as a major burden, but that some workers experienced time squeeze because of long working hours or difficulties combining different work schedules and other responsibilities. Whether or not MJH resulted in a personal burden was influenced by expectations of employers. Having rigid employers made it less easy to combine different work schedules, for instance [12]. Although the study by Bamberry et al. has provided valuable insights in experiences with MJH, it also has limitations. For instance, it was based on short interviews and participants in the study were selected randomly, which is rather unusual in qualitative research [16]. More in-depth qualitative research could provide additional insights into experiences with MJH. Furthermore, studying experiences with MJH in different countries may provide additional insights as the context in which MJH is conducted may influence experiences.

Experiences with MJH among older workers are of special importance as in many countries the statutory retirement age is increasing [17]. Consequently, the working population is ageing in many countries. On an individual level, increasing statutory retirement ages requires workers to prolong their working lives. Negative experiences with MJH, such as time squeeze, may make it more difficult for older workers to work until the statutory retirement age. On the other hand, MJH may stimulate the use of existing skills and acquisition of new skills, which could enhance sustainable employability [18]. The objective of the present study is to gain insight in experiences with MJH among Dutch workers aged 45 years and older. Because of our focus on the personal experiences of multiple job holders, in-depth interviews will be conducted.

## Methods

### Study design

The present study was part of a larger study into reasons for and experiences with MJH among older workers. A qualitative approach was chosen, because little is known about reasons for and experiences with MJH. We decided interviews rather than focus groups would be best suited to answer our research question, as previous research has indicated multiple job holders form a heterogeneous group, who may not be able to relate to each other’s accounts in a focus group. In addition‚ respondents may want to raise sensitive subjects, such as financial difficulties as a reason for MJH, which they may be more reluctant to do in a focus group.

### Participants

Participants were selected from Study on Transitions in Employment, Ability and Motivation (STREAM). STREAM is a Dutch longitudinal study among 15,118 persons aged 45–64 years at baseline (2010). The study population has been extensively described elsewhere [19]. Participants in STREAM annually fill in questionnaires on health and work, among other things. We included respondents who (1) had multiple jobs in the fifth measurement of STREAM (2015), and (2) had given permission to be contacted for future research. Participants were selected from STREAM because it enabled purposive sampling of participants using a wide range of characteristics. We aimed to include a heterogeneous group of multiple job holders, because little was known about experiences with MJH beforehand and because previous studies have indicated that multiple job holders constitute a heterogeneous group of workers [12]. Determinants of MJH may differ between workers in different age groups, and with different educational levels, and with different types of MJH, i.e. only jobs as an employee versus jobs as an employee as well as being self-employed [5, 20, 21]. Differences in determinants of MJH may be related to differences in reasons for MJH and experiences with MJH. In addition, it has been suggested that results found among a heterogeneous population are more easily generalizable than results from a homogeneous population [22]. To assure heterogeneity, purposive sampling was applied. We aimed to interview multiple job holders who were heterogeneous regarding age, gender, educational level and type of MJH (only jobs as an employee versus jobs as an employee as well as being self-employed), chronic illness (yes/no), household financial position (short of money/just adequate/money left), and the willingness to continue MJH or stop in favor of having a single job.

Potential respondents were phoned by SB to determine whether they were eligible for participation in this study, i.e. whether or not they still had multiple jobs. In total, 31 respondents were contacted between May and July 2016. Seven respondents did not answer. Four respondents did answer, but indicated they did not have time to talk on the phone. Three respondents were not willing to participate; one respondent because of personal reasons, two because of a busy work schedule. One respondent was not eligible for participation. One respondent canceled an interview appointment, because of illness. After data saturation occurred, no more potential respondents were phoned.

### Topic list

For the interviews, a topic list was made. Preliminary versions of the topic list were discussed among all authors. Before the topics were introduced to the respondents, the interviewer introduced himself and introduced the aims of the study again. In addition, respondents were asked whether they had any questions about the information letter, which was sent after the initial contact by phone. Also, their permission was asked to record the interviews. The final topic list consisted of four main topics. The first topic was related to the current work situation of the respondents. They were asked to describe the characteristics of the jobs they had at that moment. The second topic focused on the work history and the history of having multiple jobs of the respondent. The third topic was related to reasons for MJH. The fourth topic covered experiences with MJH. Respondents were asked to describe any advantages and disadvantages of MJH they experienced and why they still had multiple jobs if they mainly experiences disadvantages. The final topic list is included in Appendix 1.

### Interview setting

SB conducted all interviews, which took place between May and July 2016. During one interview, AdW was present. The first five interviews, which were part of the pilot phase of this study, which was aimed at finetuning the topic list and making a final decision on the study design. These interviews were conducted by phone. After the pilot phase, we decided that face-to-face in-depth interviews would be most suitable to answer our research objective, because they would stimulate respondents to give elaborate accounts of their experiences with MJH. Most of the face-to-face interviews were conducted at the respondents’ homes. Two interviews were conducted at a café at the request of the respondent. The interviews, as well as the expression of willingness to participate, were digitally recorded and field notes were taken. On average, interviews lasted 52 min. At the end of the interview, participants received a gift voucher worth €10,- to thank them for participating in the study.

### Analyses

Thematic content analysis was applied to analyze the data. Analysis of the data was conducted in four phases, which were derived from Braun and Clarke’s approach to thematic analysis [23]. In all phases at least two authors were involved (SB and AdW) to enhance reliability. In the first phase, SB and AdW familiarized themselves with the data. In this phase, SB read and summarized the transcripts of interviews. These summaries were then shared with AdW.

In the second phase, the transcripts were openly coded, which was deemed suitable for the present study as little was known beforehand about MJH. During the open coding process, there was an emphasis on within case analysis, i.e. thoroughly understanding each respondent’s motivation, situation, and experience. In this phase, AdW read and coded three interviews. SB and AdW discussed the codes of these interviews to determine agreement, thereby improving reliability. This phase resulted in a ‘code tree’, in which all codes were represented. The code tree was discussed among all authors.

In the third phase, building on the code tree, themes were developed. We used constant comparison between interviews (across case analyses) to find patterns in the data, thus ensuring that the themes reflected these patterns. The themes were connected in a preliminary thematic map, which was constructed by SB and discussed with AdW. During these discussions, for each preliminary theme potential ‘outliers’ were reviewed to learn why these respondents did not fit that theme. After three meetings, SB and AdW agreed on a concept thematic map.

In the fourth phase, SB reviewed the concept thematic map against the original code tree to determine whether or not the thematic map fit the data. As a result, some ‘subthemes’ were added. Adapted thematic maps were discussed between SB and AdW and again reviewed against the code tree until consensus was reached. Thereafter, the thematic map was discussed among all authors. The final thematic map is included in Appendix 2.

### Ethics

The Medical Ethics Committee of VU University Medical Center declared that no ethical approval was needed to conduct this study. All participants gave informed consent after having read an information letter regarding this study.

## Results

### Study population

Of the 15 multiple job holders we interviewed, eight were male and seven female. Four respondents had a low level of education, six intermediate and five high. Five respondents reported having a chronic health problem. Eight respondents only had jobs as an employee and seven respondents were self-employed in at least one job. Three respondents reported that their household was short of money, three that their financial situation was adequate and seven that they had money left. Twelve respondents reported whether they would rather have one job; seven said they did, and five said that they did not. Respondents worked in varying sectors of the economy, including postal services, the cleaning industry, hospitality, health care, and the financial sector. The number of jobs they had ranged from two to five. Based on the experiences of the respondents, we identified three main subgroups of multiple job holders. First, those for whom the positive consequences of MJH clearly outweighed the negative consequences. Second, workers who did not really experience any negative or positive consequences of MJH. Third, multiple job holders who mainly experienced negative consequences of MJH. In the remainder of the results section, pseudonyms will be used to refer to respondents.

### Experiences with MJH

Four main themes emerged from the interviews. Firstly, work characteristics influence respondents’ experiences with MJH. Secondly, personal context affects how work characteristics influence experiences with MJH. Thirdly, negative experiences with work characteristics often coincide with problems in personal context. Fourthly, experiences with MJH can change as part of a dynamic process.

#### Work characteristics influence experience with MJH

Most respondents mentioned that characteristics of their jobs influenced their experience with MJH. They mentioned characteristics of the combination of jobs as well as characteristics of individual jobs. Variation as a result from having heterogeneous jobs, as well as positive spill-over effects between jobs, and financial benefits resulting from working more than full-time were mentioned as combination characteristics that positively influenced experiences with MJH. Conflicts between work schedules were mentioned as negatively influencing experiences with MJH. Regarding characteristics of individual jobs, contract types that increased insecurity, e.g. temporary contracts, and a lack of social support and societal relevance of a job negatively influenced experiences with MJH.

Having a combination of jobs that varied regarding tasks and/or contacts with clients contributed to a positive experience with MJH for many respondents. For instance, John, who owned an IT-business next to being a teacher, mentioned how his business enabled him to keep programming, which he enjoyed but had become increasingly less important in his job as a teacher. William described how different the contacts with the clients of his insurance business were from the contacts with the wedding couples he spoke with as a marriage officiant, and how this contributed to his job satisfaction.

*“Regarding communication, those are two completely distinct conversations, always. One is always consultancy, more boring, more formal. And with marriages, I always interact with two people who say: ‘there’s nothing else in the world that is important right now, what do we care about work? We are getting married!’”* (William).

Other respondents noted that having similar jobs, or jobs in a similar sector of the economy, can lead to ‘spill-over’ effects, which can contribute to a positive experience with MJH. Jennifer, for instance, explained how her job as a nurse provided her with insights that she could apply in her second job as a trainer of medical professionals. In turn, her second job enlarged her network among medical professionals, through which she received information which helped her in her job as a nurse. Richard also mentioned that he often applied the knowledge he acquired in his business to his job as a civil servant, and vice versa.

Some respondents mentioned financial benefits of having multiple jobs. For those who did not have multiple jobs out of financial necessity, having extra jobs provided them with financial extras. For instance, David, who combined a full-time job with a part-time job, mentioned that the financial benefits of MJH contributed to his positive experience. Those with a job as an employee and being self-employed mentioned that being an employee provided them with income security now as well as in the future.

*“But the reality is of course that being self-employed comes with a lot of insecurity. Being a marriage officiant provides a steady income”.*

*“An employee is, of course, well taken care off. So the combination of being self-employed and an employee also kind of provides a social safety net”* (both William).

Negative consequences caused by the characteristics of the combination of jobs were also mentioned. Having multiple jobs on one day or during one week resulted in a lack of recovery time and fatigue. Susan, for instance, mentioned that she often cleaned schools in the mornings and then ran a paper route in the afternoon. She did not experience the time in between these jobs as recovery time. Linda explained how she had not had a holiday in years, in which she did not have to work for at least one employer.

*“…when it’s a quiet period at one employer, then at the other employer it will be a busy period. Yeah, for a few years now I’ve had to work during my holiday”* (Linda).

Some respondents also mentioned that conflicting work schedules caused stress and fatigue. William mentioned that he was not available for customers of his insurance company when he was at one of his other jobs. This was very stressful for him, because he perceived being available 24/7 as the unique selling point of his insurance company. Susan mentioned that sometimes she had to start very early to be able to combine the work schedules of her three jobs.

*“The combination of jobs; you sometimes have to think: ‘how am I ever going to solve this? I guess I’ll have to get up at 4 a.m. tomorrow’. That is really bizarre…”* (Susan).

Conflicts between work schedules often occurred because of inflexible working hours. Susan was only allowed to clean schools when the school was closed. Right after the schools closed, she had her paper route, which meant that she either had to clean schools before they opened or in the evening. She opted for the early mornings, because she did not want to miss out on relaxing activities in the evening. In some instances, respondents reported ‘creating’ flexibility. Charles, for instance, mentioned delivering mail too early or too late, enabling him to combine two of his three paper routes, thereby reducing his working hours without reducing his income.

Regarding individual jobs, participants most often mentioned characteristics that influenced job satisfaction, financial security, and flexibility. Having a temporary contract, a zero hour contract or being self-employed was mentioned by some respondents as a source of insecurity, but by others as a source of flexibility. Regarding job satisfaction, respondent mentioned the extent to which their job fit their identity. For example, Susan did not feel she was a ‘cleaner’ and Patricia identified as a singer, but did not get booked for enough performances to make a living, so mainly relied on her other job for income. Social support at work and perceived societal relevance of a job also influenced job satisfaction. Linda, for instance, explained that because she only worked six hours a week in one of her jobs, she did not feel connected with her colleagues. This also made her feel like she did not contribute much to the organization she worked for, and to society as a whole.

*“…I don’t get connected, I feel like I’m invisible. Very conscious of the fact that I’m just there to ‘mind the shop’, but I don’t contribute much and that reduces the fun I have at my job. I want to be able to contribute to society”* (Linda).

For some multiple job holders, their experience was exclusively influenced by characteristics of the individual jobs. For them, their experience with MJH did not differ from having one job. Karen, for instance, who combined four jobs, described that instead of a daily routine like single job holders, she had a weekly routine. Although each day of the week was different for her, each Monday, for instance, was identical. Because of this, she felt that her situation was not much different from a single job holder’s situation.

#### Personal context influences experience with MJH

Respondents’ personal context affected how work characteristics influenced their experience with MJH. The personal context included personal characteristics and reasons for MJH. Regarding personal characteristics, respondents reported that having a temporary contract was worse for older workers and those with a poor household financial situation. In addition, for some living alone meant not being able to share the burden of combining multiple jobs with other roles, and for others it meant more flexibility due to not having to take into account schedules of family members. Having a clear reason for MJH made it easier to deal with any negative consequences.

Age influenced the extent to which respondents felt confident they would be interesting for employers. As a result, it influenced the extent to which flexible work arrangements caused either stress or a feeling of autonomy. Those who felt they were less interesting to employers because of their age felt dependent on their current employers for work and income. This sometimes caused them to take on shifts they would not have taken on otherwise, which according to Barbara sometimes led her to ‘bite of more than she could chew’.

Financial position of household also strongly influenced how work characteristics affected experiences with MJH. For instance, it influenced the extent to which the flexibility provided by zero hour contracts was utilized. Respondents who were short of money or could just get by, often felt dependent on their employers for income, especially those who were breadwinner in their household. As a result, these respondents would take on shifts during their holiday, for instance.

Composition of the household also influenced respondents’ experience with MJH. Having a partner, for instance, enabled some multiple job holders to share the burden of having multiple jobs and any other roles, such as raising children. Michael, for instance, mentioned that his wife did most of the administrative tasks that resulted from owning a shop. He admitted that if it would not have been for his wife, he would have closed his shop a long time ago, because his wife was better than him at those tasks and because they were very time consuming. Some respondents also mentioned that, since they were not the breadwinner in their household, they felt less dependent on their employer for work. As a result, it was easier for them to say ‘no’ to shifts that did not fit their private life or other jobs. An exception was Elizabeth, who mentioned that not having a partner gave her more flexibility to arrange her working week how she wanted.

Some respondents mentioned that having a clear reason for MJH made it easier for them to cope with negative experiences. For instance, Robert explained that having multiple jobs enabled him to retain his family’s home, which was very important to him, since two of their children were born there and all of their children grew up there. In addition, it enabled him and his wife to raise their children the way they thought was right. As a result, he was better able to cope with his long working hours (up to 16 h per day).

“*Ultimately, the moment you think of the rewards connected to having multiple jobs, that your children can go to school nicely washed and in normal clothes and with the right attitude, then you know what you’re doing it for*” (Robert).

#### Negative work experiences and problems in personal context coincide

Most respondents who reported being dissatisfied with their work, also mentioned problems in their personal context. Often, persistent financial difficulties and financial necessity to have multiple jobs coincided with dissatisfaction with the MJH situation (see fictional case in Table 1). Even though she had three jobs, Susan, for instance, was short of money every month. These persistent financial difficulties caused problems in her private life. For instance, she continuously had to keep track of all her expenses, which drained her mentally. She also mentioned not having money to seek medical help when she needed it. These financial difficulties also negatively affected her job satisfaction.

**Table 1:** Fictional case 1: Victoria

Victoria’s (57) experience with MJH was negative. She had two jobs, as a cleaner and as a waitress, neither of which she enjoyed. Her job as a cleaner was based on a fixed hours contract, for 20 h per week. For her job as a waitress she had a temporary zero hour contract. Victoria was often tired because she experienced her jobs as physically demanding, especially for a woman her age. In addition, her tiredness was caused by long working hours; particularly on days when she worked in both jobs. On such days, she often did not have the opportunity to eat properly. However, she felt that she could not turn down those shifts, as she needed the money and did not want to risk losing her contract. The work schedules of her jobs sometimes interfered with each other and with her private life: she could not accept shifts as a waitress because of her job as a cleaner, which caused her to worry about her financial situation and prospects in the waitress job. In addition, she often did not have time, energy, or money, for her favorite hobby: swimming. In addition to her two jobs, she gave informal care to her brother. As she lived alone, she was also responsible for keeping the household running. These roles added to her tiredness and stress. Victoria felt locked into her situation, as she felt she did not have the skills and knowledge to find a full-time job that she would enjoy. Furthermore, the combination of her jobs, informal care and household work meant that she had little time or energy to search for jobs that she would like. However, she did not have the financial possibilities to reduce the working hours in her current jobs.

*“…I don’t look forward to anything anymore. I don’t have anything to look forward to anymore either. I think the work is demanding too because I don’t get anything back for it. Nothing at all”* (Susan).

Linda also described dissatisfaction with her jobs, problems in her private life and how these influenced each other. Several health problems had caused her to combine multiple part-time jobs since she did not feel that she was capable of working in a full-time job on the level she had previously worked. However, she experienced having multiple jobs as stressful. As a result, her health had deteriorated again. In addition, having low paying jobs also meant that she had had persistent financial difficulties over the last years.

On the other hand, respondents who reported having multiple jobs for other reasons than financial necessity often reported satisfaction with their jobs as well as their personal context (see fictional case in Table 2). An exception was Elizabeth. Although she admitted that having multiple jobs was essentially still a financial necessity for her, she explained that she had more jobs than necessary for her to get by. As a result, having multiple zero hours contracts did not cause her to experience income insecurity. In fact, in combination with living alone and her disposable income, these flexible work arrangements resulted in ample opportunities for her to enjoy her leisure time.

**Table 2:** Fictional case 2: Ethan

Ethan (48) experienced MJH positively. He worked as a manager for a large IT company, and part-time as a secondary school teacher. Both jobs were based on fixed hours permanent contracts. He did not start his second job because he enjoyed teaching and felt it was important to teach and enthuse teenagers about IT, rather than out of financial necessity. Ethan had been able to negotiate a high level of flexibility with both his employers, which meant that there were hardly any instances of conflicting work schedules, or conflicts between work and private life. And because most of his work weeks were identical (Tuesday through Friday he worked for the IT company, and on Mondays as a teacher) he did not feel that his situation was more burdensome than having one job. Ethan also felt his jobs reinforced each other: because he worked in IT, he was able to teach his students the newest insights. Also, encountering students who were enthusiastic about IT fueled his own enthusiasm. Because of his financial situation and impressive CV, Ethan felt that he would be able to change his work situation (e.g. quit one of his jobs to look for another) if the current situation would not meet his preferences anymore, like he had done in the past.	

#### Changes in experiences with MJH

Some respondents desired changes at some point while they held multiple jobs. Some were able to make such changes, while others felt locked into their situation. A poor household financial situation seemed to be a barrier for making changes, because of a lack of money for retraining, for instance. In addition, a lack of confidence regarding the ability to find another employer contributed to feeling locked in. Further, some respondents lacked the time and energy to look for other jobs, as they worked long hours in their current jobs.

Changes to work characteristics and personal context were made deliberately by some respondents. Richard, for instance, described how, after his wife was diagnosed with breast cancer, he realized that he had spent too much time on work. Consequently, he cut back the number of hours he worked in his business. James mentioned an example of a less deliberate change in work characteristics. He described that one of his employers asked him to change his working hours and tasks. He agreed, since in the new arrangement his working hours would be concentrated in three instead of five evenings and the job would become less physically demanding, which improved his experience with MJH.

Making changes to work characteristics and personal context was more difficult for other respondents. Patricia, for instance, mentioned that she thought that the number of hours she could work as a singer would continue to decline, partly because of her age. She also did not feel confident that she would find another full-time job she would enjoy, since she lacked education and working experience and had not been able to find such a job in the last ten years. In addition, she mentioned that persistent and increasing financial difficulties may force her to sell her car, further limiting her possibilities of finding a new job.

Linda also mentioned difficulties making changes to her work situation, mainly because her three jobs were taking up too much time and energy for her to search for a job that would match her educational level and experience. However, she had the financial possibilities to quit one of her jobs to free up time and energy that she could use to find a new, full-time job.

## Discussion

The aim of this study was to investigate the experiences with MJH among Dutch workers aged 45 years and older. Experience was defined firstly as undergoing an event and secondly as impressions of that event. Respondents described a variety of ways in which they ‘underwent’ MJH and how this influenced their impression of, or satisfaction with, having multiple jobs. Some multiple job holders had a mainly positive impression of MJH, for instance because of positive spill-over effects between jobs. Others had a predominantly negative impression, e.g. because of conflicts between work schedules. In addition, some respondents’ impression of MJH was more neutral, since they did not experience many benefits or disadvantages of MJH. Experiences with MJH were often the result of a specific combination of work characteristics and personal context. Further, negative experiences with work often coincided with an unfavorable personal context. Some multiple job holders were able to change their work characteristics to improve their experience with MJH. Others, who generally were dissatisfied with their situation, had more difficulties changing their situation.

We found that experiences with MJH ranged from positive to negative. This is partly in line with a study by Bamberry et al., who found that generally MJH was not a major burden, but that for some it did have adverse consequences [12]. Also in line with that study is our finding that work characteristics influence experiences with MJH. Inflexible working times and employers were found to negatively influence experiences with MJH in both studies [12]. This is in line with research among a general population of workers, which found that higher flexibility regarding working hours was related to higher job satisfaction and better health [24]. Among multiple job holders, low flexibility regarding working hours may be related to worse health through stress resulting from conflicts between work schedules arising from of a lack of flexibility. In contrast to Bamberry et al., we did not find that long working hours resulted in negative experiences with MJH. This may be explained by the Dutch context of the present study. In the Netherlands, namely, working part-time is more common than in Australia [25]. As a result, extremely long working hours may be less common in the Netherlands, also among multiple job holders. In addition, working long hours among multiple job holders may be voluntary more often in the Netherlands than in Australia.

This study added to the study of Bamberry that, personal context, i.e. reasons for MJH and personal characteristics such as household income and composition, may influence how work characteristics influence experiences with MJH. For instance, household financial situation influenced the extent to which zero hour contracts were perceived as a source of flexibility or financial insecurity. Further, this study added that negative experiences in one job often coincide with negative experiences in other jobs as well as with problems in the personal context. As a result, multiple job holders who were most in need of recovery time, e.g. because of the demands of their jobs, often had least opportunities to recover, e.g. because they also provided informal care. This is in line with previous research which has found that having multiple roles can cause depletion, especially if an individuals’ experience of one or more of these roles is negative [26]. Insufficient recovery time is associated with low job performance, low work engagement, and an increased risk of exhaustion [27–29].

The finding that some multiple job holders were able to change their situation when desired and others were ‘locked in’ is in line with research on job crafting and job mobility in the general population [30]. Job crafting consists of four dimensions: increasing structural job resources, increasing social job resources, increasing challenging job demands, and decreasing hindering job demands [30]. In our study, we found examples of increasing challenging job demands, e.g. acquiring supervisory responsibilities, and decreasing hindering job demands, e.g. long working hours. In line with previous research, these examples of job crafting seemed to increase job satisfaction, work engagement, and general well-being among multiple hob holders [31, 32].

Based on the results of the present study, three groups of multiple job holders could be distinguished. The first group consisted of multiple job holders who hardly experienced any positive or negative consequences of MJH. They often did not have much disposable income and continued MJH because of a clear (financial) reason. Their jobs offered some flexibility which prevented conflicts between work schedules and private life. Secondly, those whose positive experiences outweighed the negative ones. In general, these workers were financially well-off, experienced a high level of flexibility regarding working hours. They often enjoyed their jobs, and experienced positive spill-over effects between jobs. The third group consisted of workers who mainly experienced negative consequences of MJH. This group mainly consisted of single women. They reported a poor financial situation of the household and had low quality jobs [33], e.g. temporary contracts with small number of working hours, evening and weekend work, etc. Conflicts between jobs and private life often occurred. Workers in this last group often reported adverse effects of MJH, such as fatigue and stress. At the same time, they felt unable to change their situation, which augmented these adverse effects.

The findings of the present study may contribute to developing policies and interventions aimed at older multiple job holders who mainly experience negative consequences of MJH. These multiple job holders often reported not being able to find other jobs that matched their preferences. Policies stimulating and (financially) facilitating life-long learning could improve the employability of older multiple job holders [31], and their chances of finding a full-time job that better matches their preferences and abilities. In addition, these multiple job holders mentioned stress caused by conflicts between work schedules. Employers may increase flexibility of work schedules to reduce conflicts between work schedules and between work and private life. This could reduce stress and fatigue and therefore contribute to sustainable employability.

Future research is recommended to address how multiple job holders who mainly experience negative consequences of MJH, can be supported. More insight into determinants of job crafting among multiple job holders is necessary, for instance. Further, future research is recommended to study which combination of work characteristics contribute to a positive experience of MJH and increased sustainable employability, and which combinations contribute to negative experiences. Such studies can result in insights in best and worst practices and could inform policies aimed at increasing the sustainable employability as well as the ability of multiple job holders to change their situation when desired.

As this study was conducted among Dutch workers, future research should determine whether similar groups also exist in other countries, with different social security systems. In addition, this study focused on older employees. Further research is recommended to study experiences with MJH in other age groups, since some findings in this study may only apply to older multiple job holders, e.g. fear of not being interesting for employers because of older age.

A strength of the present study is the thoroughness of the analyses. During each phase at least two researchers were involved, and at the end of each phase the findings were discussed among all authors. This study also has some limitations. First, the methods and analytical perspective used in this qualitative study may have influenced our findings. It is possible that different methods, e.g. focus groups, or a different analytical perspective, e.g. a grounded theory approach, may have resulted in the identification of slightly different themes. A second limitation is that some multiple job holders we contacted did not answer, or declined because of busy work schedules. It is possible that therefore, the perspective of multiple job holders who worked extremely long hours was not sufficiently included. However, we did interview some respondents who worked very long hours and others who reported time squeeze. A third limitation of this study is that no member check was performed, which may influence the validity of the results of the current study. However, we did employ several other methods to improve validity. For instance, we selected a heterogeneous study population, to avoid missing any important perspectives on experiences with MJH. In addition, all interviews were described verbatim. Further, extensive data analysis, which consisted of several phases to allow for a within and between case analysis of the data, and the involvement of at least two experienced researchers in each phase, assured that the accounts of the interviewed multiple job holders were understood thoroughly.

## Conclusion

In conclusion, we found that some older multiple job holders mainly experience positive consequences of MJH and others mainly negative consequences. Further, some older multiple job holders do not really experience any positive or negative consequences. The personal context of multiple job holders influences how their work characteristics affect experiences with MJH. Some multiple job holders are able to change their situation if their experiences are negative, while others cannot, which further augments these negative experiences. Future research should study which work characteristics improve or reduce sustainable employability of multiple job holders and how this is affected by the personal context and the ability to adapt work characteristics and personal context.

## Appendix 1

### Topic list

Introduction

- Introduce myself (and other interviewer if she is there).
- Questions about the information letter?
- Outline of the interview.
  - About one hour.
  - Questions on current work situation, work history, reasons for multiple job holding and experiences with multiple job holding.
- Ask for permission to record the interview.
- Ask whether informed consent form is clear and whether they want to sign it.

Topic 1: multiple job holding situation at this moment

- Could you tell me something about your work and the multiple jobs you have at this moment?

[Ask about following issues if respondent does not mention them:

- Number of jobs.
- Number of working hours per job.
- Flexible work schedule?
- Working in evenings/weekends?
- Contract type (permanent or other type of contract?
- Nature of job and work.
- MPORTANT: does respondent have clear primary job and secondary job? Or a palet of different jobs?]

Topic 2: work (and multiple job holding) history

- Since when do you have multiple jobs?

[Ask about following changes if respondent does not mention them:

- Number of jobs
- Distribution of total work hours over jobs.
- Contract types.
- Social benefits.]

Topic 3: Reasons for multiple job holding

- How did multiple job holding come about for you?

[If respondent mentions financial reason:

- Was it a financial necessity for you or was it more about enjoying financial extras?
  - If necessary: can you explain why it was necessary? What was it necessary for?
  - If extras: for which extra’s did you use the extra money?

If respondent does not mention timing of transition to MJH:

- Why did you choose to take on multiple jobs when you did?

If respondent does not mention changes:

- Did anything changes regarding your reasons for MJH over time?]

Topic 4: experiences with multiple job holding

- How do you experience having multiple jobs?

[If respondent does not mention any advantages:

- Does having multiple jobs have any advantages for you?

If respondent does not mention any disadvantages:

- Does having multiple jobs have any disadvantages for you?

If respondent mentions being dissatisfied:

- Why do you still have multiple jobs?

If respondent mentions to quit multi-jobbing:

- Why? Why now? What has changed?

If respondent does not say much:

- I get the impression that you do not think it is anything special to have multiple jobs. Is that right?

If a respondent does say much:

- I get the impression that having multiple jobs has a big impact on you. Is that right? Can you tell me something more about this?].

## Appendix 2

### Thematic map

Themes regarding reasons for multiple job holding were excluded.

Main theme 1 – Work characteristics influence experiences with multiple job holding.

Theme: characteristics of individual jobs as well as of combination of jobs influence experience with multiple job holding.

Theme: characteristics of individual jobs influence experience of MJH.

Subtheme: social contacts at work influence experience with MJH.

- Due to a limited amount of working hours not much contact with colleagues; contributes to negative experience of multiple job holding.
- Due to the nature of the job not much contact with colleagues; contributes ot negative experience with multiple job holding.

Subtheme: variety in a job influences experience with MJH positively.

- Contact with a variety of people in one job contributes to positive experience of MJH

Subtheme: physically demanding jobs contribute to a negative experience of MJH.

Subtheme: having a societally important job contributes to a positive experience of MJH.

Subtheme: not working full-time in a job that fits respondents identity; as a result negative experience with MJH.

- Identify strongly with one job, but not able to work full-time in that job.
- Strong sense that current jobs do not fit identity, but no strong identification with other jobs.

Subtheme: not able to do job accurately due to limited amount of working hours; contributing to a negative experience with MJH.

Subtheme: making a job more fun/looking for new challenges in job.

Subtheme: working on set days and times contributes to a sense of routine.

Subtheme: feeling dependent on employer.

- Dependent because of zero hour contract.
- Dependent because of financial problems.
- Dependent because low self-esteem regarding changes on labor market (because of age or history of burn-out and other illnesses.

Subtheme: employers are flexible; as a result fewer conflicts between work schedules and private life.

Theme: characteristics of combination of jobs influences experiences with multiple job holding.

Subtheme: more than one job in one day ➔ time between jobs is not perceived as recovery time.

Subtheme: more than one job in one day ➔ extra commuting time.

Subtheme: having to work during holiday.

Subtheme: conflicts between jobs.

- Advantage of one job disappears because of other jobs.
- Unique selling point of business partly disappears because of other job.
- Autonomy regarding working hours prevents conflicts between work schedules.
- No conflicts because business does not interfere with employer other job.

Subtheme: due to heterogeneity of jobs, variety occurs, which contributes to positive experience of MJH.

Subtheme: jobs in the same economic sector can create positive spill-over effects.

Subtheme: combining working as an employee and being self-employed combines flexibility of being elf-employed with social security of being an employee.

Subtheme: working more than 1 fte because of multiple job holding.

- Living without financial cares.
- Less time for social activities.
- Less time for a healthy lifestyle.
- Being tired because of long working hours.

Subtheme: combination of zero hour contracts provides flexibility.

- Importance of being able to say ‘no’ to employer; otherwise: too many working hours.

Subtheme: second job provides opportunities to perform tasks no longer performed in primary job.

Main theme 2 – personal context influences relation between working characteristics and experience with multiple job holding/negative experiences in work and personal context coincide (separated in two themes in paper).

Theme: due to lack of other roles, enough time for multiple jobs and therefore limited impact.

Theme: due to help with other roles, no conflicts between work and private life.

Theme: demands of jobs are shared with others in household; as a result limited demands on respondent.

Theme: other roles (volunteer work) form distraction from long working hours.

Theme: due to negative experiences at work, difficulties enjoying activities in leisure time.

Theme: comparing current situation with previous situation or situation of others, which influences experience with multiple job holding.

Subtheme: friends have successful careers.

Subtheme: successful career in the past (before illness).

Theme: personal characteristics influence relation between work characteristics and experience with multiple job holding.

Subtheme: not needing much sleep; as a result: long working hours are not a problem.

Subtheme: discipline (I have chosen to take this job, so any negative aspects have to be accepted).

Subtheme: routine is not necessary, so a lack of a routine is no problem.

Subtheme: financial security is not necessary.

- After illness realizing that life will end some time and security is not important.

Theme: goal of MJH is clear, making disadvantages less important.

Theme: jobs are too much fun to quit, so disadvantages are just accepted.

Main theme 3 – Changes in experiences.

Theme: adjust work characteristics which are experiences negatively.

Subtheme: adjust negative sides of multiple job holding, reducing its impact.

- Working fewer hours to spend more time with family.

Subtheme: being able to change job according to preferences.

- Quitting job if you do not like it.
- Making changes in job/searching for new challenges.

Subtheme: Making sure that no conflicts between jobs occur.

- Singing a contract to prevent conflicts of interest.

Theme: advantages and disadvantages of multiple job holding are recognized after a while.

Subtheme: realizing after a while that multiple job holding has been quite demanding.

## Abbreviations

MJH: Multiple job holding
STREAM: Study on Transitions in Employment, Ability, and Motivation
